# Magnetic resonance microscopy of prostate tissue: How basic science can inform clinical imaging development

**DOI:** 10.1002/jmrs.3

**Published:** 2013-02-03

**Authors:** Roger Bourne

**Affiliations:** Discipline of Medical Radiation Sciences, Faculty of Health Sciences, The University of SydneySydney, New South Wales 2006, Australia

**Keywords:** Cancer, diffusion, magnetic resonance imaging, microscopy, prostate

## Abstract

This commentary outlines how magnetic resonance imaging (MRI) microscopy studies of prostate tissue samples and whole organs have shed light on a number of clinical imaging mysteries and may enable more effective development of new clinical imaging methods.

## Magnetic Resonance Imaging and Prostate Cancer

Although prostate cancer (PCa) is the second most common cancer killer of men in the developed world, at the present time, there is no imaging modality that provides reliable information for clinical management. The biological difficulties of imaging PCa include a complex organ structure, ubiquitous heterogeneous benign tissue proliferation, and multifocal disease. The technical problems include poor organ accessibility, magnetic susceptibility heterogeneity, and organ movement.

Although magnetic resonance imaging (MRI) has excellent soft tissue contrast relative to other clinical imaging modalities, it still does not have a major role in PCa detection, staging, or treatment management. The *least unreliable* imaging method, T_2_-weighted MRI using an endorectal coil, achieves only about 60–70% sensitivity and specificity for PCa detection, and this can be improved only by about 5–10% with supplementary diffusion-weighted imaging (DWI), magnetic resonance spectroscopy (MRS), or dynamic contrast-enhanced (DCE) MRI. MRS and DCE have the additional caveats of very long scan time and risk of nephrogenic toxicity respectively.^1^–^6^

Despite the limitations of imaging-based PCa detection, the major clinical problem is not diagnosis but management and treatment planning. In prostate disease, cancer volume is a well-established independent predictor of biochemical relapse in men who undergo surgery.^7^,^8^ Cancer volume estimated by means of the determination of core involvement is a cornerstone of all active surveillance protocols for men with presumed low-grade, low-volume disease. A major limitation in the advice clinicians can give patients who are candidates for active surveillance is the discordance that exists between what is initially believed to be low-volume disease and the actual volume of disease found on histological assessment of surgical specimens.^9^ Development of an imaging method that indicates the “true” volume of disease in men with “low-volume” disease on biopsy would revolutionize the management of PCa.

In this commentary, I describe how a series of “basic science” investigations of prostate tissue samples have shed light on three clinical imaging observations and point the way towards a reliable clinical imaging tool. All these investigations involve DWI microscopy using formalin-fixed samples of tissue taken from radical prostatectomy specimens and imaged with spatial resolution up to one hundred thousand times higher than a typical DWI examination performed in a 1.5- or 3-T scanner in vivo. I argue that supplementing clinical imaging research with basic science can expedite method development and curb the unproductive investigation of blind alleys.

## DWI as a “Direct” Detector of Prostate Cancer

Conventional medical imaging modalities have poor sensitivity and specificity for PCa detection and characterization. This is most likely because the contrast mechanisms are dependent on tissue biophysical properties that are not closely related to the microscopic tissue architecture features that are the basis of cancer diagnosis. Measurement of water diffusion-based contrast relates much more directly to histopathological cancer diagnosis because the free diffusion of water in tissue is known to be constrained by intra- and extracellular structures and cell walls.

DWI has been used extensively in the study of neural tissue, and clinical management of neural disease and injury, but has had only limited clinical application to diseases of glandular tissue such as prostate. If developed beyond its current clinical limitations, DWI may provide reliable PCa volume estimation.

Despite the absence of the long fibre tracts found in neural tissue, breast and prostate tissues have normal and pathological tissue architecture potentially well suited to DWI investigations. In normal glandular tissue, the presence of a fibrous matrix and a secretory epithelium lining a network of ductal structures provides a microscopically heterogeneous environment in which these different regions are likely to have distinctly different water diffusion behaviour that can be probed with DWI. The specific changes to this normal glandular tissue architecture that define the development of cancer are likely to be detectable as changes in water diffusion behaviour that can be characterized at the microscopic scale, but still measured at the low-resolution clinical scale with appropriately designed imaging methods.

## Apparent Diffusion Coefficient Changes Associated with Cancer Are Closely Related to Different Diffusion Properties of Epithelium and Stroma

**Figure 1:**
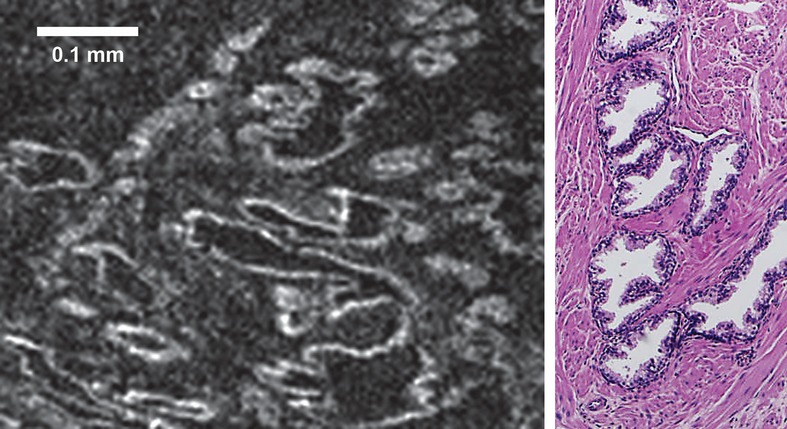
Left: Diffusion-weighted microimaging (16.4 T, 20-μm isotropic voxels) of a 3-mm-diameter core of formalin-fixed normal glandular prostate tissue demonstrates highly restricted diffusion in the approximately 15-μm-thick epithelial cell layer, moderately restricted diffusion in the fibromuscular stroma, and relatively free diffusion in the gland lumen and duct. Prior to microimaging, these diffusivity differences between microscopically adjacent different cell types were unknown. Differences in the relative partial volumes of low-diffusivity epithelial cells are the likely explanation for the clinically observed negative correlation between cancer Gleason grade and apparent diffusion coefficient (ADC). Right: H&E-stained section of normal glandular tissue.

Our initial 16.4-T MRI microscopy investigation of prostate tissue was essentially a “fishing trip” in unknown waters. The previous highest resolution study of prostate tissue was performed at 4.7 T with whole organs and 0.5-mm isotropic voxels – too large to resolve the gland structure (prostatic acini are roughly of 0.1 mm diameter).^10^ On imaging 3-mm-diameter tissue cores at 16.4 T with 40-μm isotropic voxels, T_2_*-weighted images showed the glandular structure but with low contrast. The big surprise was beautifully resolved glands in the diffusion-weighted images that revealed distinctly different water diffusion behaviours in the stroma, in the epithelial layer, and in acini and ducts (Fig. 1).^11^,^12^ A high-grade cancer sample showed no intact glands and a high density of low-diffusivity cells. Prior to microimaging, these diffusivity differences between microscopically adjacent different cell types were unknown and decreased apparent diffusion coefficient (ADC) was attributed to “increased cell density” – an assertion that hides major assumptions about the biophysical basis of restricted diffusion. These microimaging findings are thus a true “discovery” rather than a hypothesis confirmation. Changes in the relative partial volumes of stromal, epithelial, and ductal compartments are the most likely explanation for the recent clinical reports of a negative correlation between ADC and PCa Gleason grade.^13^,^14^

## Nevertheless, ADC Is a Simplistic Measure of Pathology

The single-parameter ADC estimate simplifies the potentially information-rich signal available from DWI by assuming a monoexponential model of diffusion signal decay with increasing diffusion weighting (*b*-value). Preliminary clinical investigations of diffusion signal decay suggest that a biexponential analysis may improve the accuracy of pathology assessment.^15^,^16^ In fact, non-monoexponential decay of the diffusion-weighted signal is the norm for all tissues studied to date. The biophysical basis of this complex signal decay is the subject of much speculation and some basic research, but there is a general consensus that biexponential signal decay cannot be attributed to a simple intracellular/extracellular compartmentation of water.

We have used diffusion microimaging to test a hypothesis that the biophysical basis of biexponential behaviour reported from clinical prostate MRI is the difference in diffusivities of the stromal and epithelial compartments.^17^ We found that changes in the relative partial volume of stroma and epithelium explained about 60% of the change in relative signal from the biexponential fit coefficients in normal glandular tissue. In the cancer sample we studied (Gleason 3 + 4) both high- and low-diffusivity components of the biexponential fit were significantly lower than for the normal glandular tissue – suggesting there are extensive microscopic tissue structure changes in the tumour tissue – rather than a simple proliferation of epithelial cells with “normal” diffusion properties.

These microimaging experiments confirm that the information available from diffusion measurements closely relates to the tissue structure changes associated with PCa. Can these findings be implemented in the clinic? Unfortunately, a high spatial resolution measurement of biexponential diffusion decay in vivo probably cannot produce reliable data in a clinically viable scan time, but a potential shortcut is available. Kurtosis analysis can characterize non-monoexponential decay by measurement with just two diffusion weightings (plus the unweighted reference image), rather than the 6–8 weightings required for biexponential analysis. This kurtosis approach has been successful in neural tissue,^18^,^19^ and very recently, a kurtosis analysis of prostate tissue in vivo has demonstrated improved sensitivity and specificity for cancer detection relative to conventional ADC calculations.^20^

## It Is Probably a Waste of Time Measuring Fractional Anisotropy In Vivo

Measurements of diffusion anisotropy in the prostate in vivo have produced equivocal and controversial results with widely differing fractional anisotropy (FA) values for similar tissue and no consistent correlation between pathology and FA.^21^–^24^ Some authors argue that reports of high FA are a noise artefact.^25^

Some basic science investigations have clarified the situation by characterizing the diffusion properties of prostate tissue at higher spatial resolution than can be obtained in vivo. A study of formalin-fixed radical prostatectomy specimens, performed at 4.7 T with spatial resolution (0.5 mm)^3^, obtained diffusion anisotropy data consistent with gross tissue architecture.^10^ High FA was observed in regions of primarily fibromuscular stromal tissue with the primary diffusion axis parallel to the assumed main fibre axis.

**Figure 2:**
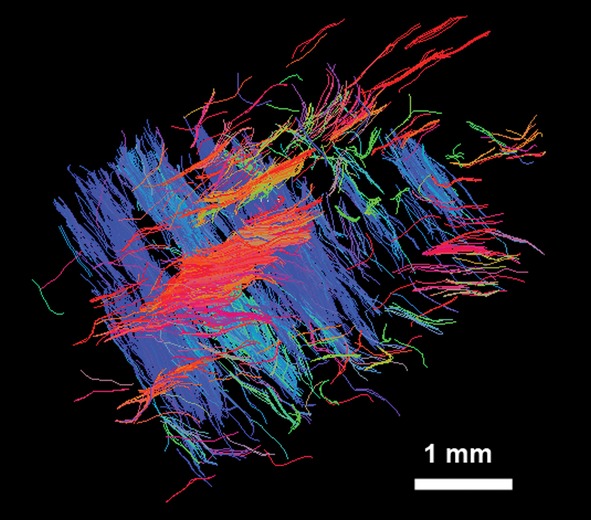
Stromal muscle fibre tracking based on diffusion tensor microimaging (16.4 T, 40-μm isotropic voxels) of a 3-mm-diameter core of formalin-fixed normal glandular prostate tissue. Colour indicates fibre direction. Despite high microscopic diffusion anisotropy, the heterogeneity of fibre directions would lead to a low estimate of fractional anisotropy in a typical voxel acquired in vivo.

We have also used DWI to characterize the fibrous structure of stromal tissue at 40-μm spatial resolution and shown that fibre tracks mapped by diffusion tensor-based tractography (Fig. 2) match myocyte orientation seen on light microscopy of the same tissue.^26^ After imaging multiple tissue samples, we concluded that the microscopic heterogeneity of stromal fibre orientation meant that attempts to use low spatial resolution diffusion tensor imaging (DTI)-based measures of diffusion anisotropy was almost guaranteed to be unreliable – as has been the case so far in clinical MRI.

Obviously, a more sophisticated measure of diffusion heterogeneity than FA is required – one that is sensitive to microscopic heterogeneity while performing a low spatial resolution (large voxel volume) measurement in vivo in a clinical scanner. One possibility is the newly developed “double wave vector” method successfully implemented for human brain tissue characterization.^27^–^29^

## Flight Testing Potential Clinical Imaging Methods

Nobody tests new aeroplane designs on the public. So, why are most ideas for new clinical imaging methods tested on patients in vivo? The multiple contradictory and inconclusive papers describing attempts to measure FA in the prostate in vivo are a good example.

MRI research performed in vivo is expensive (currently $600–$800/h in Australia), and is limited by the amount of time a patient or volunteer can reasonably be expected to lie in the scanner.

Accurate correlation of image data with tissue structure/pathology is difficult and the results are often confounded by uncontrollable experimental variables (patient and organ movement, blood flow, susceptibility artefacts, etc.). While many of these variables are present in the clinical imaging environment, their presence at the method testing stage can obscure an assessment of the *basic feasibility* of the method. If a method does not work under ideal conditions ex vivo, then there is little point in wasting public money, human resources, and volunteers' time attempting to test the method in vivo.

Obviously, this approach will only work in situations where tissue is available for imaging ex vivo. Here, we have one advantage with prostate research that partially offsets the difficulties that contribute to the current limitations of prostate MRI in vivo. Whole organs are regularly resected intact and can be imaged under well-controlled conditions ex vivo using high-field, high-resolution scanners. Imaging ex vivo is relatively inexpensive ($150/h or less), permits very long scan times, and reduces the organ shape change problems that complicate reliable correlation of in vivo imaging data with histopathology results.

**Figure 3:**
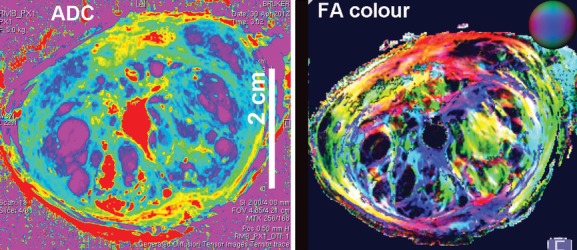
Left: Apparent diffusion coefficient map based on diffusion tensor microimaging (9.4 T, 100-μm isotropic voxels) of a whole formalin-fixed prostate. Right: Colour-coded fractional anisotropy. Note the heterogeneity of stromal fibre directions.

High-field ex vivo studies of whole organs can provide a wealth of detailed information about the way normal and pathological tissue properties affect MRI contrast. This information could be used to design clinical imaging methods that can reliably discriminate between tissue types (e.g. measure cancer grade, position, and volume) and be performed in a reasonable time in a low-field, low spatial resolution clinical system. If the proposed method works ex vivo, then it can be confidently moved to the clinical trial stage, which will include the confounding factors encountered in vivo. In this case, clinical method development can focus on addressing any in vivo confounds that reduce the known accuracy of the basic method. Figure 3 shows our first results of imaging a whole prostate at 9.4 T with (100 μm)^3^ resolution.

## How Low Can You Go?

**Figure 4:**
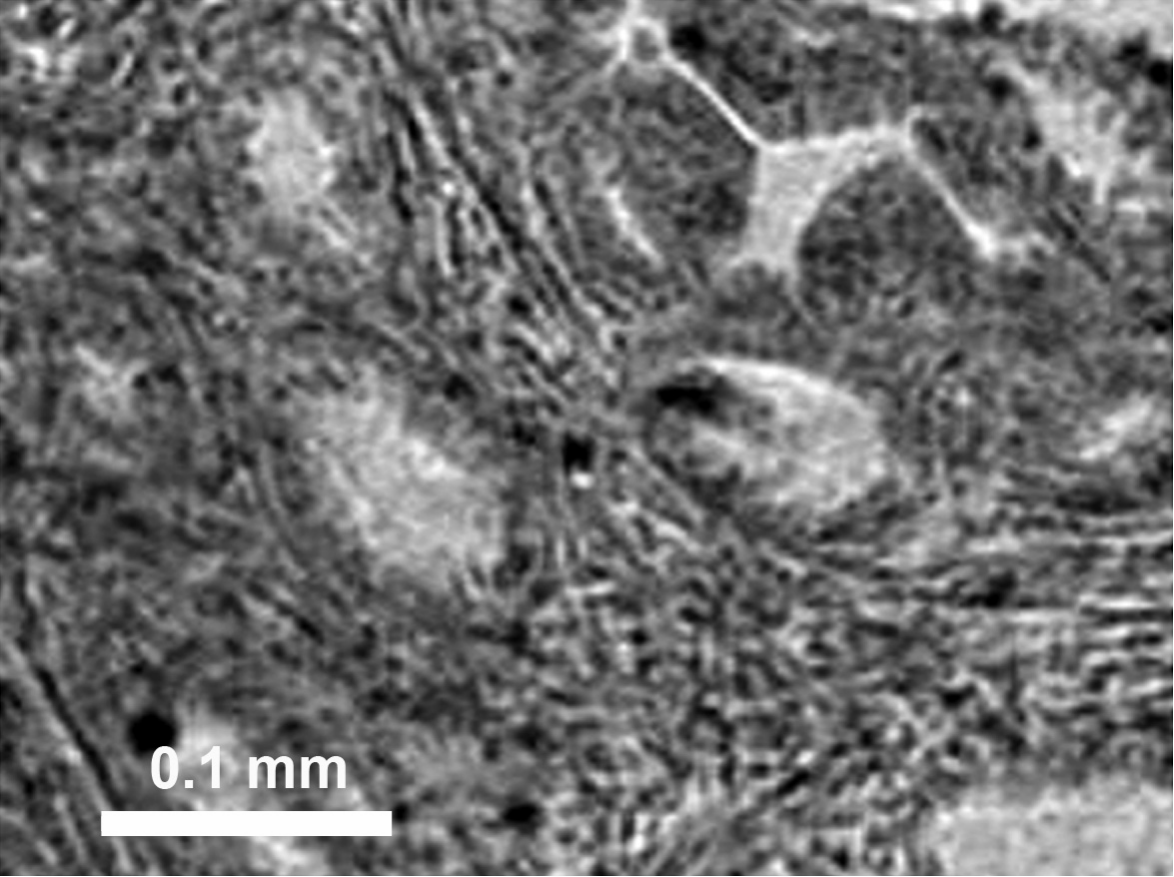
Subcellular scale magnetic resonance imaging (MRI). Ten-micrometre isotropic voxels show individual cells. Some nuclei are discernible in epithelial cells. At present, the biophysical basis of the contrast is unknown in these T_2_*-weighted images (Image courtesy of Dr Viktor Vegh, Centre for Advanced Imaging, University of Queensland).

A (40 μm)^3^ voxel contains roughly 20 cells of 15 μm diameter, so we have not yet achieved the subcellular image resolution required for complete understanding of the way cellular and tissue structure affects the diffusion of water. While this goal is many years away, there is a steady progress. Using custom-built microimaging coils in the 16.4-T scanner, our collaborators at the Centre for Advanced Imaging, University of Queensland, have recently acquired diffusion-weighted images of zebra fish embryos with 20-μm isotropic voxels and T_2_*-weighted images of prostate tissue with 10-μm isotropic voxels (Fig. 4) – possibly the highest resolution magnetic resonance (MR) image of tissue ever acquired.

## Limitations

Because of the long imaging times required for high spatial resolution measurements, most tissue samples need to be stabilized by formalin fixation. Water diffusivity in vivo is generally higher than in fixed tissue, and it is possible that diffusivity and anisotropy differences between tissue and cells types may be altered by the fixation process. Low spatial resolution comparisons of fresh and fixed prostate tissues suggest that relative changes are minor.^10^ In our studies, we found that the relative diffusivities of the biexponential fit coefficients were very similar to those found in vivo.^17^ Another advantage of imaging whole organs ex vivo is that a fresh unfixed specimen can be imaged quickly at medium resolution and then fixed and reimaged in exactly the same planes. Such experiments will enable us to fully characterize the effects of fixation.

## References

[1] Mazaheri Y, Shukla-Dave A, Muellner A, Hricak H (2011). MRI of the prostate: Clinical relevance and emerging applications. J Magn Reson Imaging.

[2] Turkbey B, Thomasson D, Pang YX, Bernardo M, Choyke PL (2010). The role of dynamic contrast-enhanced MRI in cancer diagnosis and treatment. Diagn Interv Radiol.

[3] Weinreb JC, Blume JD, Coakley FV (2009). Prostate cancer: Sextant localization at MR Imaging and MR spectroscopic imaging before prostatectomy – results of ACRIN prospective multi-institutional clinicopathologic study. Radiology.

[4] Seitz M, Shukla-Dave A, Bjartell A, Touijer K, Sciarra A, Bastian PJ, Stief C, Hricak H, Graser A (2009). Functional magnetic resonance imaging in prostate cancer. Eur Radiol.

[5] Riches SF, Payne GS, Morgan VA, Sandhu S, Fisher C, Germuska M, Collins DJ, Thompson A, deSouza NM (2009). MRI in the detection of prostate cancer: Combined apparent diffusion coefficient, metabolite ratio, and vascular parameters. Am J Roentgenol.

[6] Kelloff GJ, Choyke P, Coffey DS, Prostate cancer imaging working group (2009). Challenges in clinical prostate cancer: Role of imaging. Am J Roentgenol.

[7] Nelson BA (2006). Tumour volume is an independent predictor of prostate-specific antigen recurrence in patients undergoing radical prostatectomy for clnically localized prostate cancer. BJU Int.

[8] Hong MK (2011). Prostate tumour volume is an independent predictor of early biochemical recurrence in a high risk radical prostatectomy subgroup. Pathology.

[9] Jeldres C (2008). Validation of the contemporary Epstein criteria for insignificantprostate cancer in European men. Eur Urol.

[10] Xu JQ, Humphrey PA, Kibel AS, Snyder AZ, Narra VR, Ackerman JJH, Song SK (2009). Magnetic resonance diffusion characteristics of histologically defined prostate cancer in humans. Magn Reson Med.

[11] Bourne RM, Kurniawan N, Cowin G, Stait-Gardner T, Sved P, Watson G, Price WS (2012). Microscopic diffusivity compartmentation in formalin fixed prostate tissue. Magn Reson Med.

[12] Bourne R, Kurniawan N, Cowin G, Sved P, Watson G (2011). 16T diffusion microimaging of fixed prostate tissue. Preliminary findings. Magn Reson Med.

[13] Vargas HA, Akin O, Franiel T, Mazaheri Y, Zheng J, Moskowitz C, Udo K, Eastham J, Hricak H (2011). Diffusion-weighted endorectal MR imaging at 3 T for prostate cancer: Tumor detection and assessment of aggressiveness. Radiology.

[14] Turkbey B, Shah VP, Pang X (2011). Is apparent diffusion coefficient associated with clinical risk scores for prostate cancers that are visible on 3-T MR images?. Radiology.

[15] ShinMoto H, Oshio K, Tanimoto A, Higuchi N, Shigeo O, Kuribayashi S, Mulkern RV (2009). Biexponential apparent diffusion coefficients in prostate cancer. Magn Reson Imaging.

[16] Mulkern RV, Barnes AS, Haker SJ, Hung YP, Rybicki FJ, Maier SE, Tempany CMC (2006). Biexponential characterization of prostate tissue water diffusion decay curves over an extended b-factor range. Magn Reson Imaging.

[17] Bourne R, Kurniawan N, Cowin G, Chowdhury S, Sved P, Watson G, Stait-Gardner T, Price W (2012). Biexponential diffusion decay in formalin fixed prostate tissue: Preliminary findings. Magn Reson Med.

[18] Zhuo JC, Xu S, Proctor JL, Mullins RJ, Simon JZ, Fiskum G, Gullapalli RP (2012). Diffusion kurtosis as an in vivo imaging marker for reactive astrogliosis in traumatic brain injury. Neuroimage.

[19] Jensen JH, Falangola MF, Hu CX, Tabesh A, Rapalino O, Lo C, Helpern JA (2011). Preliminary observations of increased diffusional kurtosis in human brain following recent cerebral infarction. NMR Biomed.

[20] Rosenkrantz AB, Sigmund EE, Johnson G, Babb JS, Mussi TC, Melamed J, Taneja SS, Lee VS, Jensen JH (2012). Prostate cancer: Feasibility and preliminary experience of a diffusional kurtosis model for detection and assessment of aggressiveness of peripheral zone cancer. Radiology.

[21] Haker SJ, Szot Barnes A, Maier SE, Tempany CM, Mulkern RV (2005).

[22] Sinha S, Sinha U (2004). In vivo diffusion tensor imaging of the human prostate. Magn Reson Med.

[23] Gurses B, Kabakci N, Kovanlikaya A, Firat Z, Bayram A, Ulug AM, Kovanlikaya I (2008). Diffusion tensor imaging of the normal prostate at 3 Tesla. Eur Radiol.

[24] Gibbs P, Pickles MD, Turnbull LW (2006). Diffusion imaging of the prostate at 3.0 tesla. Invest Radiol.

[25] Reinsberg S, Brewster J, Payne G, Leach M, deSouza N (2005).

[26] Bourne R, Kurniawan N, Cowin G, Sved P, Watson G (2012). Microscopic diffusion anisotropy in formalin fixed prostate tissue: Preliminary findings. Magn Reson Med.

[27] Koch MA, Finsterbusch J (2011). Towards compartment size estimation in vivo based on double wave vector diffusion weighting. NMR Biomed.

[28] Lawrenz M, Finsterbusch J (2011). Detection of microscopic diffusion anisotropy on a whole-body MR system with double wave vector imaging. Magn Reson Med.

[29] Shemesh N, Cohen Y (2011). Microscopic and compartment shape anisotropies in gray and white matter revealed by angular bipolar double-PFG MR. Magn Reson Med.

